# Effective deep learning approaches for predicting COVID-19 outcomes from chest computed tomography volumes

**DOI:** 10.1038/s41598-022-05532-0

**Published:** 2022-02-02

**Authors:** Anthony Ortiz, Anusua Trivedi, Jocelyn Desbiens, Marian Blazes, Caleb Robinson, Sunil Gupta, Rahul Dodhia, Pavan K. Bhatraju, W. Conrad Liles, Aaron Lee, Juan M. Lavista Ferres

**Affiliations:** 1 AI for Good Research Lab, Microsoft, Seattle, WA USA; 2 Intelligent Retinal Imaging Systems, Pensacola, FL USA; 3 Department of Ophthalmology, University of Washington, Seattle, WA USA; 4 Department of Medicine and Sepsis Center of Research Excellence, University of Washington (SCORE-UW), Seattle, WA USA

**Keywords:** Viral infection, Computed tomography, Computer science

## Abstract

The rapid evolution of the novel coronavirus disease (COVID-19) pandemic has resulted in an urgent need for effective clinical tools to reduce transmission and manage severe illness. Numerous teams are quickly developing artificial intelligence approaches to these problems, including using deep learning to predict COVID-19 diagnosis and prognosis from chest computed tomography (CT) imaging data. In this work, we assess the value of aggregated chest CT data for COVID-19 prognosis compared to clinical metadata alone. We develop a novel patient-level algorithm to aggregate the chest CT volume into a 2D representation that can be easily integrated with clinical metadata to distinguish COVID-19 pneumonia from chest CT volumes from healthy participants and participants with other viral pneumonia. Furthermore, we present a multitask model for joint segmentation of different classes of pulmonary lesions present in COVID-19 infected lungs that can outperform individual segmentation models for each task. We directly compare this multitask segmentation approach to combining feature-agnostic volumetric CT classification feature maps with clinical metadata for predicting mortality. We show that the combination of features derived from the chest CT volumes improve the AUC performance to 0.80 from the 0.52 obtained by using patients’ clinical data alone. These approaches enable the automated extraction of clinically relevant features from chest CT volumes for risk stratification of COVID-19 patients.

## Introduction

The Coronavirus Disease (COVID)-19 pandemic has generated an unprecedented global health response in an effort to reduce transmission and mortality. Since the early stages of the pandemic, computed tomography (CT) chest imaging has been a valuable assessment tool. Experts have developed an understanding of COVID-19-associated chest CT findings, which include ground-glass opacities (GGOs), consolidation, bilateral involvement, and peripheral and diffuse distribution^1^. Early chest CT abnormalities may be absent or nonspecific; however^2,3^ and deep learning models have been developed to identify subtle imaging features and distinguish COVID-19 pneumonia from normal findings or other pneumonias^4^. Deep learning models have also been trained to predict outcomes such as hospitalization, intubation, and/or mortality based on CT imaging data^5,6^. Xiao et al.^6^ created a deep learning model using multiple instance learning and ResNet34 to analyze CT images from 408 COVID-19 patients for severe disease. The model was able to predict severe disease in a subgroup analysis of patients who presented with non-severe symptoms. Other studies have combined CT features with other clinical data to predict outcomes. Zhang et al.^7^ used volumetric lung lesion features extracted from a segmentation model along with clinical metadata. They show that the pulmonary lesions were most predictive for progression to severe disease, followed by clinical parameters related to lung function, age, and fever on admission. Wang et al.^5^ used CT images to train a model to diagnose COVID-19 and risk-stratify patients for severe disease.

**Figure 1:**
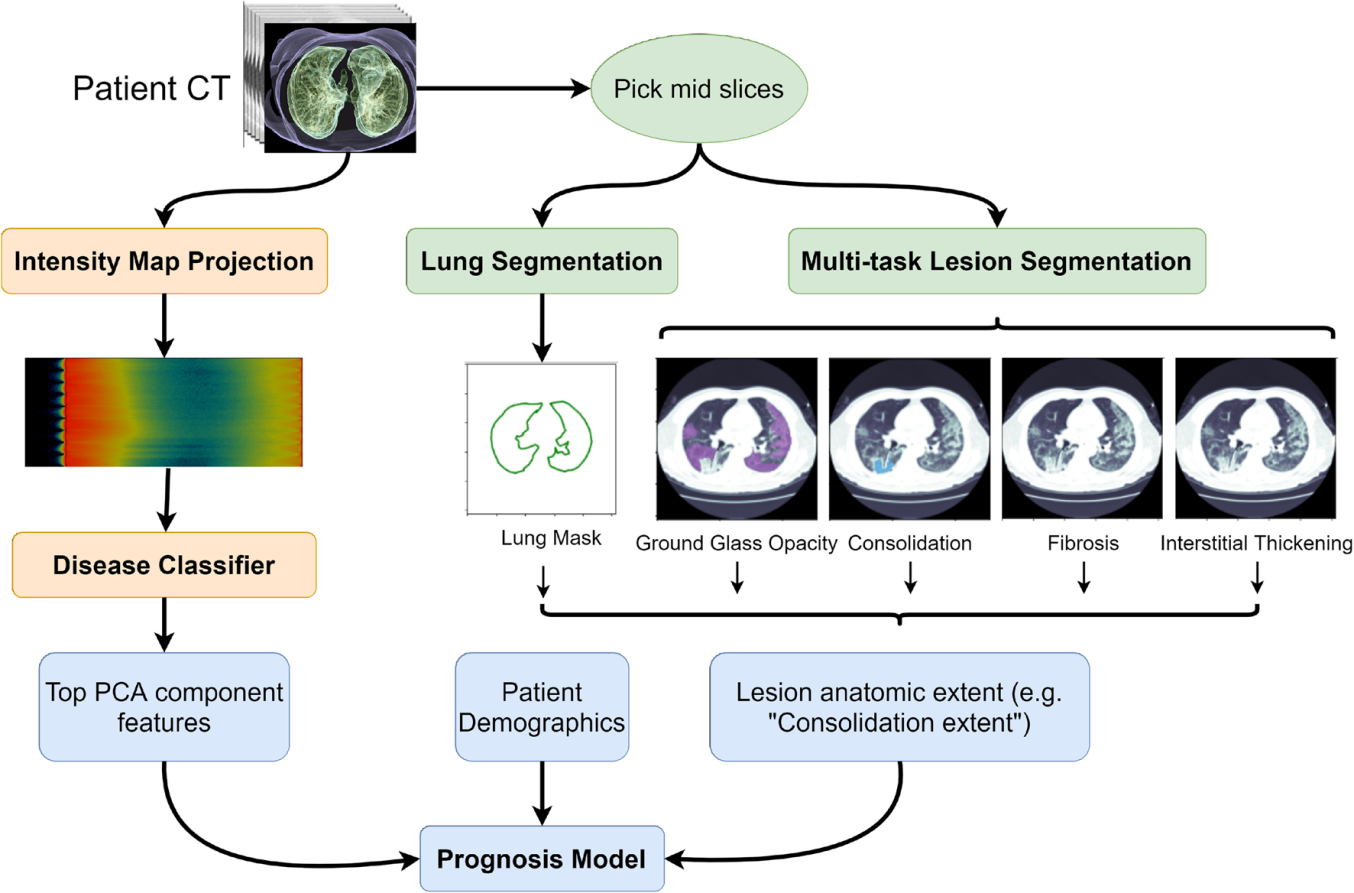
CT-based machine learning pipeline for COVID-19 prognosis. The left side of the figure (orange) represents the intensity map projection and disease classifier presented in the "Methods" section. The right side if the figure (green) shows the use of multitask semantic segmentation to obtain lesion anatomic extent features. Features obtained from the disease classifier, lesion anatomic extent features, and patient’s demographics are then used for mortality prediction using a prognosis model (blue).

Despite these advances, machine learning approaches with volumetric CT data remain challenging due to a large number of slices in relatively few patients (the curse of dimensionality). Many advances rely on using each slice as independent training examples. In this study, we explore deep learning approaches for extracting clinically relevant features from chest CT volumes using limited data. Figure 1 shows an overview of the CT-based machine learning pipeline we propose. We develop a novel method for aggregating the chest CT volume into a 2D representation of global features that can distinguish COVID-19 pneumonia from other viral pneumonia and normal chest CT volumes, with state-of-the-art performance. Furthermore, we present a multitask model for joint segmentation of different classes of pulmonary lesions present in COVID-19 infected lungs with the goal of extracting local, highly relevant features. We then create prognostic models using the extracted features together with patient demographic data, comparing the performance of models using different combinations of relevant data to predict mortality. The overall goal of this work is to enable automated extraction of relevant features from chest CT volumes that can be incorporated with clinical data for risk stratification of COVID-19 patients. We follow the guidelines for applying artificial intelligence to medical imaging proposed by Mongan et al.^8^ in terms of providing modeling details to ensure reproducibility.

## Related work

### Classification

 There are already some published studies on CT-based COVID-19 diagnosis systems^9,10^. Many researchers^3,4,6,7,11^ have proposed different feature extraction approaches to exploit the power of CT scans for COVID-19 diagnosis and prognosis. Zhang et al^7^ and Al-Karawi et al^11^ created a COVID-19 analysis framework on a dataset comprising 4,154 patients that can separate COVID-19 from other basic pneumonia. Zhang et al^7^ process CT scans in a two step fashion. Li et al^4^ built an AI framework for COVID-19 identification on a dataset comprising of 3,322 subjects. Li et al^4^ developed a 3D deep learning model for the diagnosis of COVID-19, referred to as COVNet. COVNet takes as input a series of CT slices, extracts features from each slice using a ResNet50 backbone, and finally combines obtained features using the max-pooling operation. Obtained features are then used to generate a classification prediction for the entire CT scan.

### Segmentation

 In addition to diagnostic models, several prediction models have been proposed based on an assessment of lung lesions. There are three typical classes of lesions that can be detected in COVID-19 chest CT scans: ground-glass opacity (GGO), consolidation, and pleural effusion^12,13^. Imaging features of the lesions, including shape, location, extent, and distribution of involvement of each abnormality, have been found to have good predictive power for mortality^14^ or hospital stay^15^. These features, however, are mostly derived from the delineated lesions, and so depend heavily on lesion segmentation. Harrison et al^16^ showed that a deep learning-based segmentation beats a specialized methodology in cases with interstitial lung maladies. Following the same idea, automatic lung lesion segmentation for COVID-19 has been actively investigated in recent studies. In another recent study by Chaganti et al^17^, a lesion segmentation model supported by the 3D-Dense U-Net architecture was proposed and trained on CT scans of a mixture of 160 COVID-19, 172 viral infection, and 296 interstitial lung disease patients. Although the lesion masks weren’t compared voxel-to-voxel, the volumetric measures of lesions, like percentage of opacity and consolidation, showed a high correlation between automatic and manual segmentation. These studies suggest that lesion features could be a useful biomarker for COVID-19 patient severity assessment.

### Prognosis

 Prognostic models based on automated lesion segmentation features and other CT derived features have been developed. Xiao et al^6^ created a deep learning model using multiple instance learning and ResNet34 to analyze CT images from 408 COVID-19+ patients for severe disease. The model was able to predict severe disease in a subgroup analysis of patients who presented with non-severe symptoms. Other studies have combined CT features with other clinical data to predict outcomes. Zhang et al^7^ uses volumetric lung lesion features extracted from the segmentation model along with clinical metadata. They combine the features using a tree-based ensemble model to predict disease severity (defined as ICU admit, intubation, or death), and show that the pulmonary lesions were most predictive for progression to severe disease, followed by clinical parameters related to lung function, age, and fever on admission. Wang et al^5^ used CT images to train a model to diagnose COVID-19 and risk-stratify patients for severe disease. The first model segmented the lung region then performed a non-lung area suppression operation to hide any non-pulmonary features further. The prognostic model was first trained on a large dataset of 4106 CT-EGFR+ lung cancer patients to predict EGFR mutation status (wild type vs. mutant) based on the segmented lung region, and subsequently trained to detect COVID-19 in a separate dataset of 1266 patients. A set of three COVID-19 features were extracted and combined with clinical data (age, sex, comorbidities) to classify patients into high-risk/low-risk categories. The patients classified as high risk by the model were shown to have significantly longer hospital stays.

## Methods

### Datasets

#### CC-CCII classification dataset

 For the classification experiments, we used a clean version (duplicate and X-ray scans removed) of the CC-CCII dataset, a large CT dataset from the China Consortium of Chest CT Image Investigation(CC-CCII), consisting of CT images from retrospective cohorts. It consists of CT images from Sun Yat-sen Memorial Hospital and Third Affiliated Hospital of Sun Yat-sen University, The First Affiliated Hospital of Anhui Medical University, West China Hospital, Guangzhou Medical University First Affiliated Hospital, Nanjing Renmin Hospital, Yichang Central People’s Hospital, and Renmin Hospital of Wuhan University. CT imaging was performed as a part of patients’ routine clinical care, including CT images from COVID-19 and other viral pneumonias (Pneumonia group). Pneumonia group consists of common types of viral pneumonia, including adenoviral, influenza, and para-influenza pneumonia.

The classification dataset we used in this paper, the CC-CCII^7^ dataset, consists of a total of 514,103 CT slices from 2,471 patients. It includes 156,070 slices from 839 COVID-19+ patients, 159,700 slices from 874 viral pneumonia patients, and 95,459 slices from 758 normal patients. The splits were performed at the CT level with a Train/Test/Validation ratio of 90%/5%/5%. As patients might have undergone multiple scans, for patient classification, we used the highest severity prediction against the highest severity label.

#### CC-CCII pulmonary lesions segmentation dataset

 For the pulmonary lesions segmentation experiments, a set of CT slices were manually segmented slices from the CC-CCII dataset. The segmentation labels were selected as relevant pathological features for distinguishing COVID-19 from other common pneumonia. The annotation included lung field and five commonly seen categories of lesions, including lung consolidation, ground-glass opacity(GGO), pulmonary fibrosis, interstitial thickening, and pleural effusion. Segmentation results were annotated and reviewed by five senior radiologists with 15 to 25 years of experience. The full dataset includes a slice segmented for each one of the 1302 available CT scans along with the corresponding polygons outlining each pulmonary lesion present in the slice. 293 slices showed pulmonary fibrosis lesions, 294 slices were obtained from patients within the first week of being diagnosed with COVID-19, 45 slices came from patients after having mild COVID-19, 201 slices were obtained from patients after severe COVID-19, and 489 were from patients with intermediate COVID-19 severity. The severity level was determined based on the size and type of pulmonary lesions as defined by Zhang et al.^7^. Mild was defined as less than three GGO lesions of size less than 3 cm; intermediate was defined as a lesion area more than 25% of the entire lung field; and severe was defined as a lesion area more than 50% of the entire lung field. The segmentation slices were assigned with random probability of 80%, 10%, and 10% to one of three distinct sets: training (1035 slices), validation (134 slices), and test (133 slices). This was done at the severity level to keep similar severity proportions among the sets. The partitions were disjoint at the study (CT scan) level. Best models were selected using the performance on the validation set and were evaluated on the held-out test set.

#### CC-CCII prognosis dataset

 For the prognosis model, a set of 701 scans containing 61,810 CT slices from 136 COVID-19 patients (130 surviving, 6 deceased) at hospital admission in the CC-CCII dataset were labeled as severe (defined as a lesion area more than 50%of the entire lung field) and were not used for any of the other experiments. The data from these COVID-19 patients were combined with available demographics information (age and sex only) to create a set of 105 patients (101 surviving, 4 deceased) with demographics and imaging feature data for the prognosis experiments.

#### Stony Brook University (SBU) prognosis dataset

 To show how our imaging models generalize and improve prognosis, we further tested our models on a larger dataset, with a completely different demographic background, from Stony Brook University (SBU)^18^. This dataset includes CT scans (1.25 mm & 5 mm) from 288 unique COVID-19 positive patients. The full CT volumes are available for 241 of those patients. 205 of these patients survived and 36 died. The data from these 241 COVID-19 patients were combined with available demographics information (age and sex only) to create a dataset for the prognosis experiments.

#### Ethics statement

 This study was conducted in accordance with the Declaration of Helsinki. CT images were collected from cohorts from the China Consortium of Chest CT Image Investigation (CC-CCII), which consists of Sun Yat-sen Memorial Hospital and Third Affiliated Hospital of Sun Yat-sen University, Anhui Medical University, West China Hospital, Nanjing Renmin Hospital, Yichang Central People’s Hospital, and Renmin Hospital of Wuhan University. Informed consent was obtained from all subjects and/or their legal guardian(s), and Institutional Review Board approvals were obtained by all of the institutions. The data are open sourced at: http://ncov-ai.big.ac.cn/download?lang=en. We received additional segmentation labels from CC-CCII for certain pulmonary lesions not included in the open source release. The CT images from Stony Brook University are hosted by TCIA^19^ and the data are open sourced^18^.

### Intensity map projection

We first created a solution for global feature extraction. The proposed method allowed the creation of a fixed-size 2-D global representation of any 3-dimensional CT volume by aggregating individual CT slices/planes’ intensity histogram as horizontal entries to create an intensity map.

**Figure 2:**
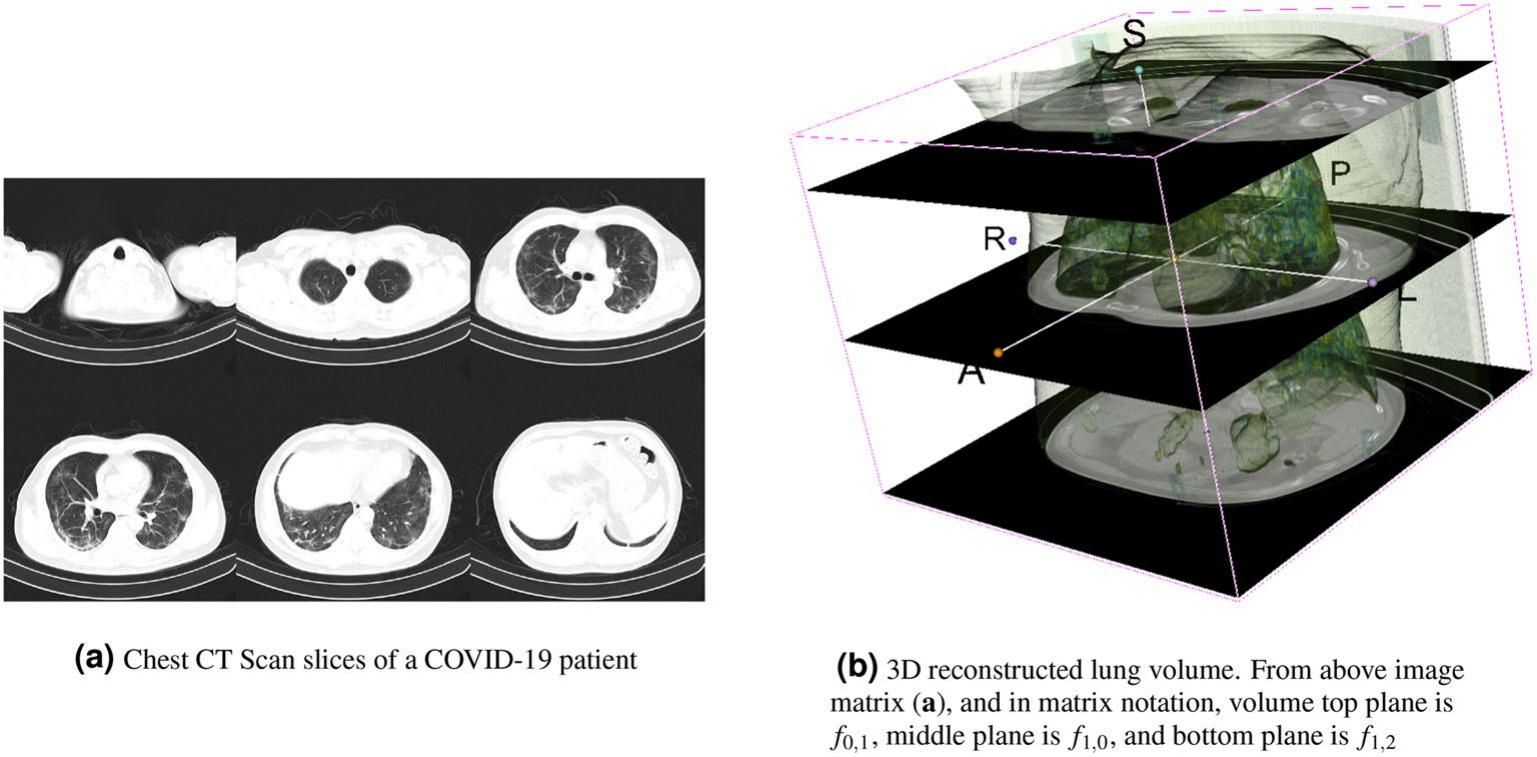
CT scan slices and lung volume reconstruction.

#### Texture analysis

 Texture analysis methods have proven helpful in describing medical images. The oldest and most widely used texture analysis technique is the intensity histogram. It counts the number of occurrences of intensity values for all pixels of an image and builds a histogram from the cumulative entries. Its output is also a set of features/frequencies.

#### Occlusion mask

 Sometimes, partly visible objects on an image are blocked by other objects located in the foreground. This phenomenon is called *occlusion*. One can think of lesions (GGO, consolidations, fibrosis, …) as occlusion objects that are masking the “normal” background image.

Let $f_{k}$, with $k=0,\ldots ,N-1$, be *N* images, or planes, of the same size. Then, the occlusion mask set of the $f_{k}$ planes with respect to a given *label* function $\phi :{\mathscr {X}}\rightarrow {\mathbb {Z}}$ is $\text {occ}_\phi \{f_k\}_{k=0}^{N-1}$. In other words, for any pixel *x*, the label $\phi (x)$ determines which one of the multiple pixel values $\{f_k\}_{k=0}^{N-1}$ actually appears in the final image $\left( \text {occ}_\phi \{f_k\}_{k=0}^{N-1}\right) (x)$ at precisely *x*.

#### Composite image

Let $\text {occ}_\phi \{f_k\}_{k=0}^{N-1}$ be an occlusion mask set with *N* planes. Then, the expected value of the histogram transform of a composite image (the original image) is a linear convex combination of the histograms of each individual plane, *i.e.*

1 $$\begin{aligned} \sum _{k=0}^{N-1}\lambda _k{\mathscr {F}}_k(x, y) \end{aligned}$$

where $\sum _{k=0}^{N-1}\lambda _k=1$, and $\lambda _k\ge 0$ for all $k=0,\ldots ,N-1$. Here, the ${\mathscr {F}}_k$ are the histograms of the $f_k$ planes, for $k=0,\ldots ,N-1$ (See^20^, Theorem 1).

#### Volume decomposition

The projections identify with the CT slices (see Fig. 2a) when there are enough slices. Otherwise the slices are stacked up, resized vertically and finally re-sampled horizontally to have the needed number of horizontal planes. Let *V* a 3D-volume composed of *M* horizontal planes $p_j$. Put $V=p_0\oplus p_1\oplus \cdots \oplus p_{M-1}$, with $\oplus$ being the image stacking operation (see Fig. 2b).

Finally, under the operator $\oplus$, the histogram of a volume *V* is the sum of the histograms of its composing planes $p_j$, with the convention that lesions are mapped to the occlusion mask sets $\text {occ}_{\phi ^j}\{f_k^j\}_{k=0}^{N_j-1}$ for $j=0,\ldots ,M-1$.

### Intensity map of CT volumes

Based on Eq. (1) and volume decomposition, we propose to aggregate individual CT slices/planes for creating the *intensity map* as shown in Algorithm 1. The algorithm sets the intensity map as the unit of prediction for a CT scan while preserving the natural ordering of the slices/planes in the final 2D intensity map. We used the ImageNet pre-trained InceptionResnetV2 as the main classification model. Training was performed over the global set of resampled (if the number of slices doesn’t match) 256 slices volumes, each row representing a typical gray-scale 256 bins intensity histogram. For the optimizer we used Nadam with a learning rate equal to 0.0001, and categorical Cross-Entropy was used as the loss function. All classification models were trained using Tensorflow and Keras on 4 GPUs.

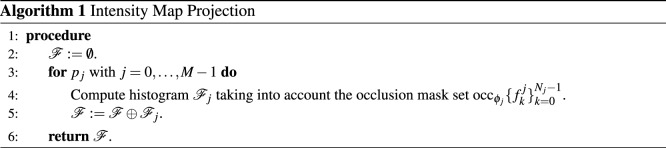



### Obtaining pulmonary lesion features for COVID-19 prognosis model

We let $\left( x_{n}\right) _{n = 1}^{N}$ represent a set of training CT slices. Each slice $x_n$ is associated with a corresponding lung contour mask $l_n$. Depending on whether the slice $x_n$ shows the presence of a pulmonary lesion it will be also associated to the corresponding lesion masks element of the set $lesions \in \{ggo_n, cl_n, fl_n, it_n\}$. For each pixel (*i*, *j*) in the CT slice we aim to assign a label $l_n$ when the pixel is located within the lung contours: $ggo_n$ for pixels where ground glass opacity is present, $cl_n$ for consolidation, $fl_n$ for every pixel showing signs if pulmonary fibrosis, and $it_n$ for pixels where interstitial thickening is present. The lesion coverage for a particular lesion can then be computed as the ratio between the total amount of pixels showing signs of the lesion and the total of lung contour ($l_n$) pixels present on the slice. It is important to notice that a pixel (*i*, *j*) in a CT slice image can be associated with multiple lesions.

#### Multi-decoder segmentation network

 We proposed a “multi-task multi-decoder” segmentation network inspired by the U-Net^21^ architecture where the encoding part is shared among the different pulmonary lesion tasks with independent decoding heads. We referred to it as “multi-task segm. net”, a U-Net-like multitask network architecture where both encoder and decoder parameters are shared among the different segmentation tasks. See Figure A.2 describing the proposed network architecture in the Appendix section B.

All training slices in the segmentation dataset were divided by 255 to get values in a range from zero to one. Resulting slices are pre-processed for zero mean and unit variance using mean (0.481456) and (0.398588) and standard deviation calculated over the entire dataset. Both “multi-task multidecoder segm. net” and “multi-task segm. net” networks can be trained end-to-end using gradient-based optimization. The full criterion is described in Eq. (2), where $\alpha _{GGO}$, $\alpha _{cl}$, $\alpha _{fl}$, and $\alpha _{it}$ are hyper-parameters. Since the tasks are very imbalanced, we used the ratio of the number of slices not showing the lesion over the number of slices showing that particular lesion as the corresponding alpha value (1.29, 1.53, 2.9, 7 respectively in our experiments). $L_{GGO}$, $L_{cl}$, $L_{fl}$, and $L_{it}$ can be any standard segmentation loss as Jaccard or binary cross-entropy (BCE). For our experiments we used weighted BCE with weights 0.3 for background and 0.7 for lesion prediction, since often the lesions cover a very small region of the slices.

All segmentation models were trained using PyTorch on 4 GPUs. All layers but Batch Normalization were initialized using PyTorch’s default initialization method. Batch Norm weights were initialized using a normal distribution. Refer to the Appendix section B.1 for more implementation details.

2 $$\begin{aligned} L_{multitask} = \alpha _{GGO} * L_{GGO} + \alpha _{cl} * L_{cl} + \alpha _{fl} * L_{fl} + \alpha _{it} * L_{it} \end{aligned}$$

We report performance results using mean intersection over Union (mIoU), a standard metric for semantic segmentation^22^.

3 $$\begin{aligned} mIoU = (1/n_{cls}) \sum _{i}n_{ii}/ (t_i + \sum _{j}n_{ji} - n_{ii}) \end{aligned}$$

where $n_{ij}$ is the number of pixels of class i predicted to belong to class j, there are $n_{cls}$ different classes, and $t_i = \sum _{j}n_{ji}$ is the total number of pixels of class i. The test models are selected based on the highest mIoU performance on the validation set.

#### Lung contour segmentation

 All segmentation slices have labels for lung contours and most standard semantic segmentation networks can segment the lung very accurately (the lung segmentation model had 94.09% mean IoU performance). For our pipeline, we used the U-Net architecture from Ronneberger et al^21^.

#### Lesion anatomic extent estimation

 A lesion anatomic extent was computed at the patient level as the percentage of the lung coverage by the lesion. The lung area was obtained using the lung segmentation model and the pulmonary lesion area was obtained from the multitask segmentation model predictions applied to multiple slices from the middle of the CT (3 to 5 slices) and averaged out lesion sizes. For patients with multiple CT scans available, the final lesion score was obtained by averaging the scan’s lesion scores. The anatomic extents of these pulmonary lesions were used as features for the prognosis model presented in the following step.

### Prognosis model

We compared the performance of five different machine learning models for mortality prediction in COVID-19 patients. For these models, we used demographic information alone, intensity map classification alone, segmentation alone, and combinations of classification and segmentation approaches combined with demographic information, in order to determine which model most accurately predicted mortality. As there were very few deaths recorded, we applied sub-sampling on the majority class (survivors) to balance the dataset. We experimented with Multi-tree XGBoost, Random Forest, Extra randomized tree, and Logistic regression models, with multiple stratified splits. Among these models, the Extra randomized tree was most successful for predicting mortality using a combination of different feature sets. To explore the global feature set from the classification pipeline, we examined two separate cutoffs for features by PCA: the top 3 and the top 18 features. The extra randomized tree classification model was trained using Scikit-Learn python library.

## Experiments and results

### Diagnosis results on CC-CCII dataset

For each CT scan, we created an intensity map representation following the methodology described in the previous section. The intensity maps can then be used as input to a classification model for diagnosis. Table 1 shows the achieved performance in both validation and test sets from the CC-CCII classification dataset using a *k*-fold Cross-Validation procedure ($k=10$).

On a per-patient basis, the overall accuracy of the InceptionResnetV2 classifier trained using our intensity map projection is 95.3% (95% CI 92.0–98.7) in the validation set and 96.0% (96% CI 92.864–99.136%) in the test set.

**Table 1:** Per-Patient Validation/Test results.

Performance	Per-Patient			Per-Scan		
	Pneumonia	COVID-19	Normal	Pneumonia	COVID-19	Normal
Accuracy (%)	98.0/98.0	95.3/97.3	97.3/96.7	97.6/98.2	98.7/98.0	98.2/98.0
Area under ROC	97.0/98.0	96.0/98.5	96.5/95.5	99.0/100.0	100.0/100.0	100.0/100.0
Specificity (%)	100.0/98.0	94.0/94.0	99.0/99.0	98.0/98.0	99.1/98.6	98.7/99.0
Sensitivity (%)	100.0/98.0	94.0/97.0	99.0/99.0	97.0/98.5	98.0/97.0	96.7/95.3
F1 Score (%)	96.9/97.0	93.3/96.1	95.9/94.8	96.8/97.5	98.2/97.2	96.7/96.3

We compare our approach to Zhang et al.^7^ which reported overall accuracy in validation and test sets of 92.49%/89.92%, with COVID-19 accuracy of 92.49%/90.70%, sensitivity of 94.9%/92.51%, specificity of 91.13%/85.92%, and ROC of 98.0%/97.1% at the scan level. On a per-scan basis, Zhang et al.^7^ showed area under ROC in validation and test sets of 96.7%/96.8% for patients with pneumonia, 98.0%/97.1% for COVID-19 patients, and 99.5%/99.9% for normal patients.

Comparing Table 1 per-scan with results from Zhang et al.^7^, our method achieved higher AUC value than the baseline method for the three classes Pneumonia/COVID-19/Normal. COVID-19 sensitivity/specificity/ROC scores are also higher, showing the usefulness of our proposed representation. Moreover, our method uses a single model and does not rely on lung or lesion segmentation.

### Multi-decoder segmentation results

The next step is to perform semantic segmentation using our proposed multi-decoder segmentation network on the CT slices of COVID-19 patients. Lesion segmentation results will later be used to obtain each lesion anatomic extent.

####  Evaluation metrics

Not all types of lesions are present in a patient CT slice. In our training set 75% of the slices presented consolidation, 87% of the slices included 1 or more GGO, but only 29% and 10% of the slices included pulmonary fibrosis and interstitial thickening respectively. In those cases, we assume that background is the only class and assign mIoU of 1 to models not predicting that class. This makes this metric not very informative for certain lesions. Hence, we also report Global mean IoU (GIoU) as the mean IoU calculated only over slices where the lesion of interest is present. For all segmentation experiments we report the average performance and corresponding standard deviation after three training runs using different random data splits.

**Table 2:** Lesion Segmentation Performance on CC-CCII Dataset.

Method	Ground-Glass		Consolidation		Fibrosis		Thickening		
	mIoU (%)	GIoU (%)	mIoU (%)	GIoU (%)	mIoU (%)	GIoU (%)	mIoU (%)	GIoU (%)	Num. of Params
Ground Glass Segm. Net.	72.81 ± 0.07	55.94 ± 2.40	−	−	−	−	−	−	3.72 M
Consolidation Segm. Net.	−	−	81.24 ± 1.80	83.14 ± 6.01	−	−	−	−	3.72 M
Fibrosis Segm. Net.	−	−	−	−	89.88 ± 2.62	94.52 ± 7.28	−	−	3.72 M
Thickening Segm. Net.	−	−	−	–	−	−	**100.00**	**100.00**	3.72 M
Multi-task Segm. Net.	68.21 ± 3.50	53.01 ± 3.50	**92.75** ± **5.92**	**90.79** ± **8.73**	**91.10** ± **2.80**	95.34 ± 2.54	99.61 ± 0.32	**100.00**	3.72 M
Multi-task multi-decoder Segm. Net.	**73.08** ± **2.59**	**74.225** ± **0.95**	80.84 ± 0.98	84.22 ± 3.16	90.21 ± 2.15	**95.52** ± **3.58**	**100.00**	98.84 ± 1.96	7.78 M

Significance values are in bold.

**Figure 3:**
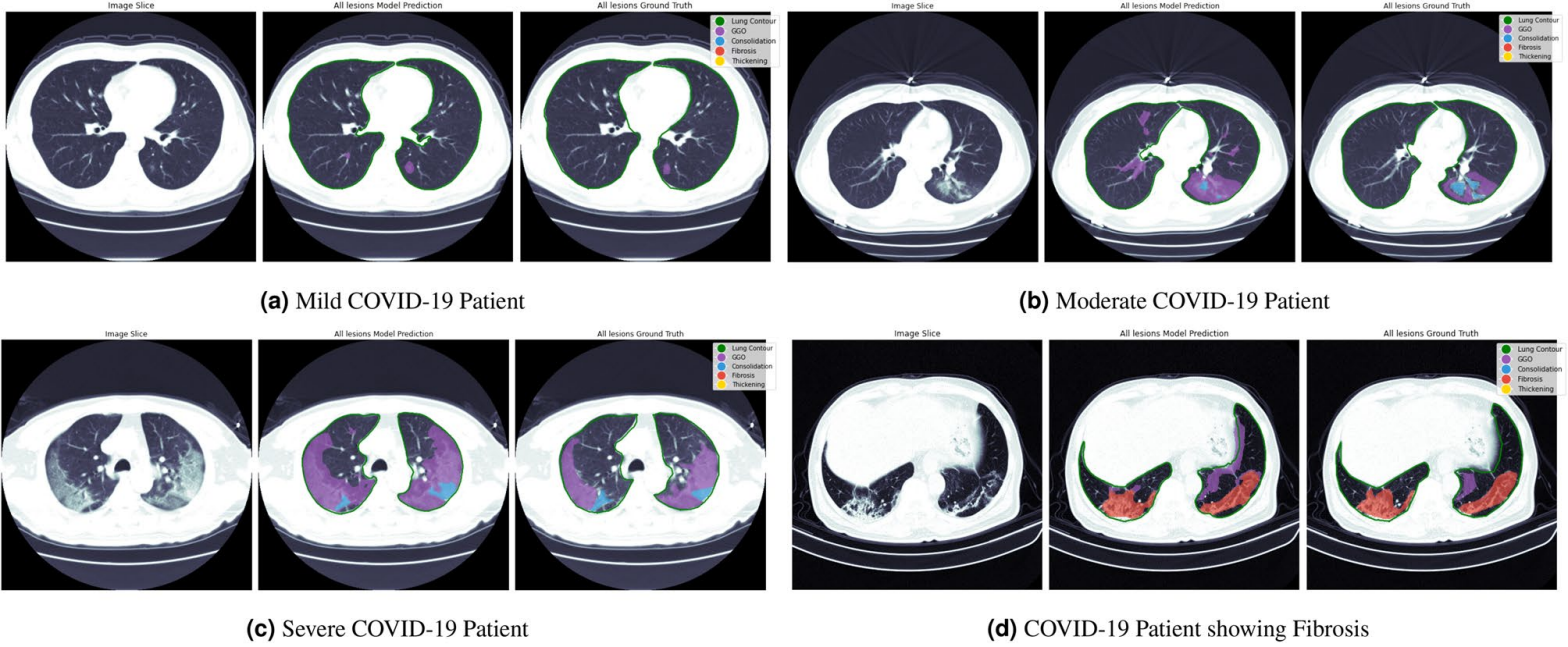
Qualitative results from our proposed lung segmentation and multitask segmentation network on COVID-19 patients presenting different levels of disease severity. For every sub-figure the first image represents the input CT slice image, the second image represents our model prediction from all different lesions and the last image represents the segmentation ground truth obtained from expert radiologists. (**a**) Model predictions on a patient with mild novel COVID-19 pneumonia with CT findings of GGO (purple), (**b**) Model predictions on a patient with moderate novel COVID-19 pneumonia with CT findings of both GGO (purple) and consolidation (blue), (**C**) Model predictions on a patient with severe novel COVID-19 pneumonia CT findings of both GGO (purple) and consolidation (blue), (**d**) Model predictions on a patient presenting severe pulmonary fibrosis (red) and GGO (purple).

Table 2 shows the quantitative performance of our proposed approach compared to using individual models. Multitask models show better performance for all pulmonary lesions even when the number of trainable parameters was the same as the parameters of individual models. The performance improvement is even more noticeable in the low data regime as is shown in the supplementary document where models were trained using 50 percent of the training data (the number of available test slices for fibrosis and thickening is very small and might not accurately reflect model’s performance). Figure 3 shows qualitative results for the “multi-task multi-decoder segm. net” on the test set. Model’s predictions closely align with the masks generated by expert radiologists.

### Prognosis results

#### CC-CCII prognosis

 We performed prognosis experiments following the leave one participant out cross-validation (LOOCV), an extreme version of *k* -fold cross-validation where *k* is set to the number of examples in the prognosis dataset described in Section 2.5 using the prognosis model previously described. Results are shown in Table 3. We use standard deviation to calculate the errors, which gives us the variability of the sample means over 10 iterations. The demographics alone (age and gender) achieved an AUC of 0.52 ± 0.01. To explore the global feature set from the classification model, we obtained top 3 and the top 18 PCA components from the features activation of the second last layer of the classification model, with the top 3 explaining 50% of the variance. These PCA components are used as input features to the prognosis model. The model using the top 18 PCA features performed poorly compared to using the top 3 features alone. Interestingly, using local data from the segmentation pipeline alone achieved similar performance to using demographics data alone. However, by including the segmentation features with the demographics, the AUC improved the AUC to 0.71, suggesting that imaging features encoded clinically relevant features for mortality prediction. We apply the ordinary least square statistical model to calculate the p-value for each feature of the prognosis model. The p-value for demographics (age and gender) showed up as statistically more significant than that of the CT segmentation and top 3 PCA features. Among those of the CT segmentation and top 3 PCA features, the feature fibrosis was the most statistically significant. The highest performing model used a combination of patient demographics, the top 3 PCA features from the classification model, and the segmentation features, for an AUC of 0.80 and 0.04 standard error. Prognosis results on all 136 severe COVID-19 patients are explained in the Appendix section D.

#### SBU prognosis

 To further generalize our models, we use a dataset from SBU with a bigger patient base of 241 patients (205 surviving, 36 deceased) and different demographic distribution. We used our prior trained imaging models to extract imaging features from the CT scans of this dataset (following the same steps as we did for CC-CCII dataset). Then we combined the patient demographics (age and sex only) with the extracted imaging features. We performed prognosis experiments following the leave one participant out cross-validation (LOOCV), as we did for the CC-CCII dataset above. Results are shown in Table 4. We use standard deviation to calculate the errors, which gives us the variability of the sample means over 10 iterations. The demographics alone (age and gender) achieved an AUC of 0.64 ± 0.03. To explore the global feature set from the classification model, we obtain the top 3 PCA components from the features activation of the second last layer of the classification model, with the top 3 explaining 90% of the variance. These PCA components are used as input features to the prognosis model. Interestingly, using local data from the segmentation pipeline alone achieved slightly better performance compared to using demographics data alone. Including the segmentation features with the demographics, the AUC improved further to 0.76, suggesting that imaging features encoded clinically relevant features for mortality prediction. The highest performing model used a combination of patient demographics, the top 3 PCA features from the classification model, and the segmentation features, for an AUC of 0.81 and 0.03 standard deviation. We apply the ordinary least square statistical model to calculate the feature importance. The demographics (age and gender) shows up with slightly higher predictive power than the CT segmentation and top 3 PCA features. The fibrosis anatomic extent shows up as the CT feature with the most predictive power.

**Table 3:** CC-CCII Prognosis Results with Leave One Out cross-validation.

Input feature sets	# features	Accuracy	F1	AUC	Precision	Recall
CT classifier features (top 18 PCA)	18	0.42 ± 0.13	0.31 ± 0.19	0.38 ± 0.04	0.11 ± 0.14	0.13 ± 0.17
CT classifier features (top 3 PCA)	3	0.76 ± 0.13	0.62 ± 0.08	0.62 ± 0.06	0.16 ± 0.15	0.17 ± 0.06
Segmentation model features	3	0.76 ± 0.26	0.62 ± 0.12	0.62 ± 0.13	0.20 ± 0.17	0.19 ± 0.21
Segm. features + CT (top 3 PCA)	6	0.86 ± 0.16	0.68 ± 0.07	0.69 ± 0.06	0.30 ± 0.22	0.29 ± 0.19
Patient demographics	2	0.68 ± 0.07	0.51 ± 0.02	0.52 ± 0.01	0.03 ± 0.02	0.15 ± 0.10
Patient demographics + CT (top 18 PCA)	20	0.71 ± 0.13	0.59 ± 0.21	0.58 ± 0.09	0.05 ± 0.03	0.14 ± 0.23
Patient demographics + CT (top 3 PCA)	5	0.77 ± 0.27	0.63 ± 024	0.62 ± 0.06	0.06 ± 0.15	0.17 ± 0.06
Patient demographics + Segm. features	5	0.88 ± 0.04	0.71 ± 0.40	0.71 ± 0.03	0.26 ± 0.40	0.24 ± 0.10
Patient demographics +	8	**0.91** ± **0.03**	**0.74** ± **0.02**	**0.80** ± **0.04**	**0.34** ± **0.02**	**0.33** ± **0.07**
CT (top 3 PCA) + Segm. features						

Significance values are in bold.

**Table 4:** Stony Brook University Prognosis Results with Leave One Out cross-validation.

Input feature sets	# features	Accuracy	F1	AUC	Precision	Recall
CT classifier features (top 3 PCA)	3	0.78 ± 0.01	0.20 ± 0.03	0.65 ± 0.01	0.25 ± 0.04	0.16 ± 0.03
Segmentation model features	3	0.78 ± 0.02	0.62 ± 0.05	0.66 ± 0.02	0.27 ± 0.09	0.02 ± 0.03
Segm. features + CT (top 3 PCA)	6	0.84 ± 0.06	0.69 ± 0.03	0.69 ± 0.02	0.32 ± 0.04	0.29 ± 0.01
Patient demographics	2	0.73 ± 0.08	0.61 ± 0.03	0.64 ± 0.03	0.33 ± 0.04	0.38 ± 0.12
Patient demographics + CT (top 3 PCA)	5	0.78 ± 0.01	0.64 ± 0.04	0.76 ± 0.01	0.29 ± 0.04	0.20 ± 0.03
Patient demographics + Segm. features	5	0.88 ± 0.01	0.69 ± 0.40	0.76 ± 0.03	0.49 ± 0.02	0.50 ± 0.01
Patient demographics +	8	**0.92** ± **0.01**	**0.75** ± **0.04**	**0.81** ± **0.03**	**0.69** ± **0.01**	**0.59** ± **0.03**
CT (top 3 PCA) + Segm. features						

Significance values are in bold.

## Discussion

We developed two methods for automated extraction of clinically relevant features from chest CT images in the setting of limited data and combined these features with demographic information to develop a prognosis model for COVID-19 outcomes. First, the intensity map projection created 2D representations of 3D CT volumes, which can then be used in a COVID-19 diagnostic model without the need for lesion segmentation. Next, the multitask segmentation model identified four pulmonary lesions specific to COVID-19 infection while computing the location and extent of each type of lesion. Taken together, these two approaches provide a method for identifying 3D pulmonary pathology data from a CT scan that can be combined with other clinical data to improve predictions about COVID-19 outcomes. We thus created a prognosis model that analyzed the most relevant CT imaging findings in combination with demographic information to achieve more accurate outcome predictions than models based on demographics, intensity mapping, or segmented features alone.

Improving the accuracy of prognosis models is critical in the setting of a pandemic. Early screening with a reliable prognosis model could reduce the burden on the hospital system, by identifying patients who could recover safely at home. Many studies have developed prognostic models for COVID-19, but a recent systematic review found that many were either poorly reported and/or at risk for bias^23^. One source of bias is the lack of available clinical data. CT imaging models are often limited by the small number of COVID-19 positive patients in the available datasets, and are further limited to analyzing only slices that contain pre-segmented lesions^9^. Our intensity map projecting method enhances global disease feature extraction from CT volumes by translating 3D information from the entire lung region into a 2D map. The resulting model performed better at COVID-19 diagnosis than a model trained on individual CT slices. COVID-19 pneumonia can be difficult to differentiate from other pneumonias in the early stages of disease, so analyzing the entire lung volume may enhance the model’s ability to detect pathology specific to COVID-19 infection. The multi-task segmentation model, however, allowed for simultaneous assessment of key localized COVID-19 pulmonary lesions and was able to take advantage of related information between regions of interest, outperforming segmentation models designed to assess individual lesions. We provide a small set of guidelines to follow while testing our models on new data in section C of the supplementary document.

Relevant imaging data can be difficult to extract and integrate with other types of clinical data when building prognosis models^24^. Our methods for extracting the features from CT data that are most specific for COVID-19 facilitates integration of such information with clinical data. The results of our prognosis model experiments demonstrate how adding the most relevant imaging data improved the performance of the prognostic model. The highest performing model used a combination of patient demographics, the top three features from the classification model (based on intensity mapping), and the segmentation features. Interestingly, using local data from the segmentation pipeline alone achieved similar performance to using demographics data alone. However by including the segmentation features with the demographics, the AUC improved, suggesting that imaging features encoded information that was clinically relevant for mortality prediction. These results suggest that both the intensity mapping approach and the segmentation approach extracted complementary clinical information from the CT volumes which, when considered together, are useful for predicting mortality. The intensity mapping provided a global representation of features from the entire volume, while the segmentation model contributed information about localized but highly relevant features. While the accuracy of the best model in Table 4 is above 90%, it is important to note that the best performing model had relatively low precision and recall. The relative importance of these features are important for understanding the disease process but further work would need to be done before this model could be used in a clinical context.

Clinicians rely on both imaging data and clinical information to assess patients and predict disease course. The most accurate prognostic models for COVID-19 outcomes will rely on information that is most specific to risk for severe COVID-19 disease. In the setting of limited patient data, it is challenging to prevent deep learning models from associating less clinically significant or even unrelated data with specific outcomes. The CC-CCII prognosis dataset used for testing includes a limited number COVID-19 deceased patients. Performing a similar analysis using a larger and more balanced dataset might be worthwhile once more data becomes available. In this study, we demonstrate an approach that combines two methods for identifying relevant pulmonary findings associated with COVID-19 disease that are useful for predicting clinical outcomes. Ideally, these approaches will help to enable rapid triage of newly infected patients, allowing for early intervention to prevent severe disease and better manage clinical resource allocation.

## Supplementary Information

Supplementary Information.
