# Supplementary material for: Effective deep learning approaches for predicting COVID-19 outcomes from chest computed tomography volumes
Source: Sci Rep. 2022 Feb 2;12:1716. doi: 10.1038/s41598-022-05532-0 (PMC8810911; doi:10.1038/s41598-022-05532-0)
Supplement: Supplementary file 1 — Supplementary Information. [file 41598_2022_5532_MOESM1_ESM.pdf]

A Supplementary Details about Intensity Mapping

As an example, Fig. A.1 is the calculated intensity map of the data shown in Fig. 2. Red color means low intensity, while blue color means high intensity. COVID-19 maps are bluer than Pneumonia/Normal maps because they host on average more high-intensity pixels than the average normal plane.

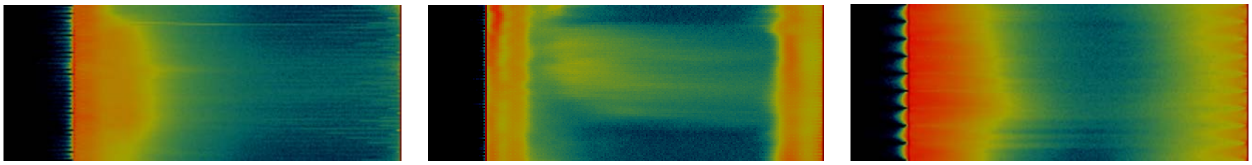

(a) COVID-19+ scan **(b)** Pneumonia scan **(c)** Normal scan

Figure A.1. Intensity maps. Top: Normal, Middle: Pneumonia, Bottom: COVID-19+. Red color means low intensity, blue color means high intensity, and black no intensity.

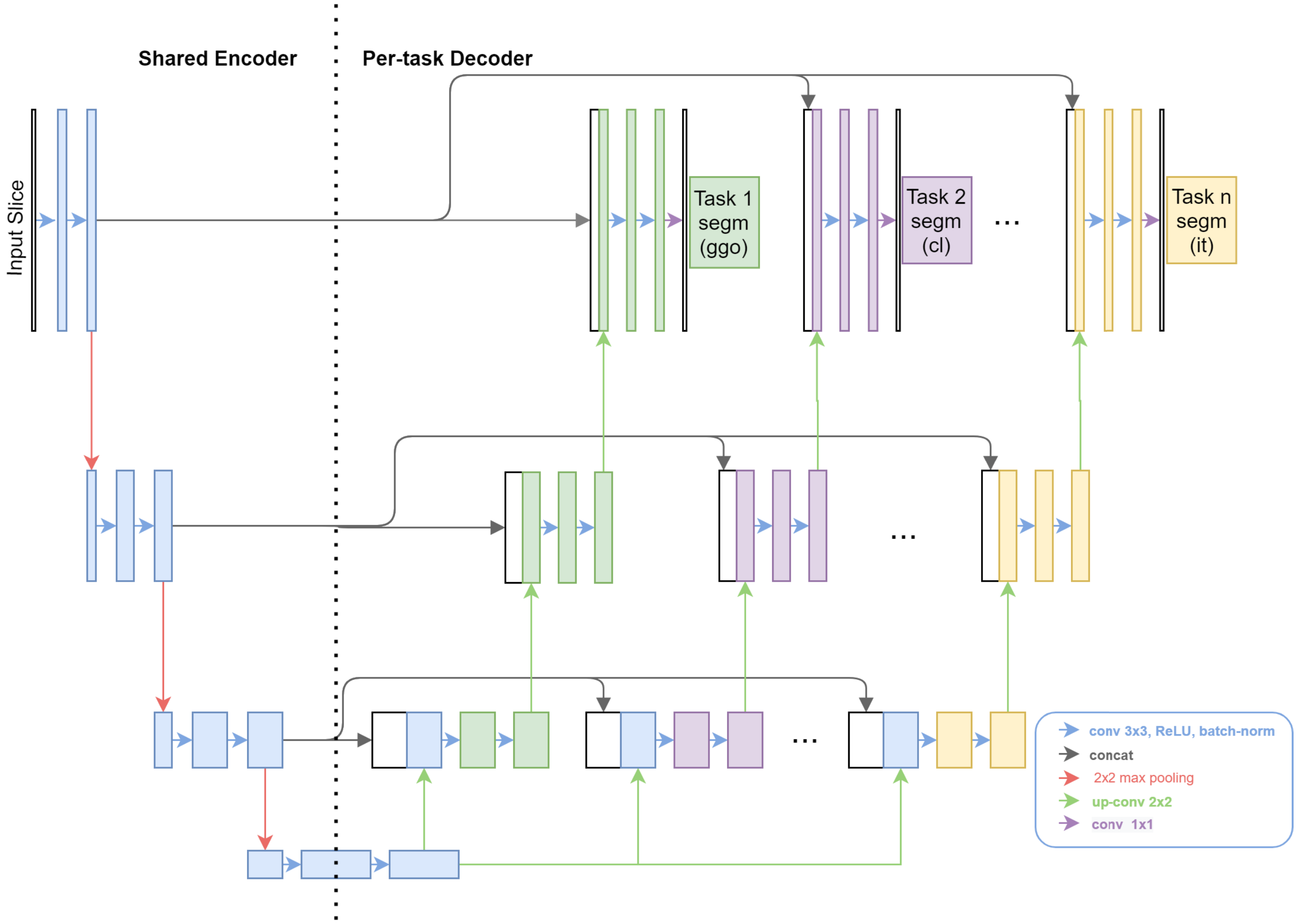

Figure A.2. Diagram of our proposed multitask segmentation network (Best seen in color).

B Supplementary Details Related to Multitask Segmentation Network Architecture

Figure A.2 shows a detailed diagram of the proposed network architecture. It is a U-Net[21] like segmentation network with shared encoder and a decoder per class. There is flexibility in the depth of the network and the number of filters per layer. It is also possible to share parameters across the different tasks on the decoder as shown on the experiments in the main document.

Table B.1. Lesion Segmentation Performance 50 percent of data

	Ground-Glass		Consolidation		Fibrosis		Thickening	
**Method**	**mIoU (%)**	**GIoU (%)**	**mIoU (%)**	**GIoU (%)**	**mIoU (%)**	**GIoU (%)**	**mIoU (%)**	**GIoU (%)**
Ground Glass Segm. Net.	67.31	51.16	- -	- -	- -	- -	- -	- -
Consolidation Segm. Net.	- -	- -	74.32	77.27	- -	- -	- -	- -
Fibrosis Segm. Net.	- -	- -	- -	- -	**100.0**	**100.0**	- -	- -
Thickening Segm. Net.	- -	- -	- -	- -	- -	- -	76.37	76.91
Multi-task multidecoder Segm. Net.	**67.62**	**51.73**	**77.21**	**82.32**	**100.0**	**100.0**	**83.50**	**84.29**

Table D.2. CC-CCII Prognosis Results on all patients with Leave One Out cross-validation

**Input feature sets**	**# features**	**Accuracy**	**F1**	**AUC**	**Precision**	**Recall**
Segm. features + CT (top 3 PCA)	6	0.88 ± 0.03	0.69 ± 0.05	0.69 ± 0.02	0.36 ± 0.27	0.27 ± 0.18

B.1 Segmentation Models Implementation Details

Lung segmentation. To obtain the lung contour model we trained several U-Net models using resized to 512×512 input CT slices with different depths and number of input filters. We used the Adam optimizer with a batch size of 24. All networks were trained from scratch with a learning rate of 0.001. We decay the learning rate by 10% on plateaus with patience of 5 epochs. The lung segmentation network was trained for 20 epochs. Weighted binary cross-entropy was used as the loss function. The best performing model on the validation set was picked as the final one.

Multitask segmentation of pulmonary lesions. Both “multi-task multidecoder segm. net" and “multi-task segm. net" networks were trained end-to-end using Adam optimizer. The loss function used for training is defined in Equation 2. For α_1, α_2, α_3, and α_4 we use the ratio of the number of slices not showing the lesion over the number of slices showing that particular lesion as the corresponding alpha value. For L_{GGO}, L_{cl}, L_{fl}, and L_{it} we used weighted binary cross-entropy with class weights 0.3 for background and 0.7. Both “multi-task multidecoder segm. net" and “multi-task segm. net" were trained from scratch with a learning rate of 0.001. We decay the learning rate by 10% on plateaus with patience of 5 epochs and train until convergence. A single training sample consists of a CT slice image along with a binary mask per task. Slices were normalize to be zero-mean and show unit variance using mean and variance from the training split.

B.2 Multitask network with limited training data

Table B.1 shows the performance of the different segmentation models trained using 50% of the available training data. The improvement in performance from the multi-decoder segmentation network is greater in the low data regime.

C Considerations while testing these models on external data

Our models were developed for 8 bits imagery. You should consider standardizing your CT slices to range between 0 and 1. Percentile standardization is useful if the intensities are long tail distributed. Fine-tuning the models on a subset of your data is highly encourage if feasible. Otherwise, it would be necessary to align the distribution of your scans to the ones we used for training (histogram matching would be a way to accomplish that). Images should normalize to be zero-mean and unit variance. Code with our model architectures implementation accompanied with instructions on how to use it will be available on GitHub at: `https://github.com/microsoft/covid19-ct-segmentation`

D Testing prognosis models on all severe COVID-19 patients

In this section, we explore whether just using CT imaging features from a larger number of severe COVID-19 patients could outperform prognosis results from combined demographic and CT imaging features from a smaller subset of severe COVID-19 patients.

We ran the prognosis models on all 136 severe COVID-19 patients, using only image feature from the classification and segmentation models, and skipping the missing demographic information. We performed prognosis experiments following the leave one participant out cross-validation (LOOCV), an extreme version of k-fold cross-validation where k is set to the number of examples in the prognosis dataset described in Section 2.5 using the prognosis model previously described. Results are shown in Table D.2. As seen from the numbers, the prognosis results on only imagining features on full vs. subset of severe COVID-19 patients are comparable.
